# Electrical characterization of single nanometer-wide Si fins in dense arrays

**DOI:** 10.3762/bjnano.9.178

**Published:** 2018-06-25

**Authors:** Steven Folkersma, Janusz Bogdanowicz, Andreas Schulze, Paola Favia, Dirch H Petersen, Ole Hansen, Henrik H Henrichsen, Peter F Nielsen, Lior Shiv, Wilfried Vandervorst

**Affiliations:** 1 IMEC, Kapeldreef 75, B-3000 Leuven, Belgium; 2 Instituut voor Kern- en Stralingsfysika, KU Leuven, Celestijnenlaan 200D, B-3001 Leuven, Belgium; 3 Department of Micro- and Nanotechnology, Technical University of Denmark, DTU Nanotech Building 345 East, DK-2800 Kgs. Lyngby, Denmark; 4 CAPRES A/S, Scion-DTU, Building 373, DK-2800 Kgs. Lyngby, Denmark

**Keywords:** critical dimension metrology, electrical characterization, finFET, micro four-point probe, sheet resistance

## Abstract

This paper demonstrates the development of a methodology using the micro four-point probe (μ4PP) technique to electrically characterize single nanometer-wide fins arranged in dense arrays. We show that through the concept of carefully controlling the electrical contact formation process, the electrical measurement can be confined to one individual fin although the used measurement electrodes physically contact more than one fin. We demonstrate that we can precisely measure the resistance of individual ca. 20 nm wide fins and that we can correlate the measured variations in fin resistance with variations in their nanometric width. Due to the demonstrated high precision of the technique, this opens the prospect for the use of μ4PP in electrical critical dimension metrology.

## Introduction

The transition from planar to three-dimensional transistor architectures such as the fin field-effect transistor (finFET) [1] has raised the need for measuring the electrical properties of nanometer-wide conducting features [2]. Recently, it has been shown that the micro four-point probe (μ4pp) technique, which is commonly used for sheet resistance measurements on blanket materials or relatively large pads (larger than 80 × 80 µm^2^) [3–5], provides a solution to this requirement [6]. The μ4pp technique was demonstrated to provide (sheet) resistance measurements in single fins without the need for dedicated Kelvin resistor or transmission line structures [7]. However, the results demonstrated in [6] focused on isolated fins whereby the fin pitch was larger than the contact size of the μ4pp electrodes such that only one single fin was contacted at a time. Intuitively, this suggests that the technique developed therein fails when trying to measure dense structures where a fin pitch smaller than the apparent contact size of the electrodes is used (see below in Figure 2). In that case, the μ4pp technique appears to be of limited value in routine semiconductor manufacturing where state-of-the-art chips use much smaller fin pitches [8].

In this paper, we describe further developments of the μ4pp technique, as implemented by the CAPRES A300 tool, which enable the electrical characterization of single nanometer-wide fins in dense fin arrays (pitch < 200 nm) with high precision and repeatability. First, we describe the general concept of how to establish and control the electrical contact between the metallic (Ni-coated) μ4pp electrodes and the semiconducting (Si) fins. Next, we show that, by carefully controlling this process, the electrical contact can be confined to one single fin such that the resistance of individual fins in dense arrays can be measured with a high precision. Finally, we use the technique to determine the electrical resistance of individual fins in a dense array and we demonstrate that the measured resistance correlates with the geometrical width of the fins, as measured with transmission electron microscopy (TEM). Due to the demonstrated high precision, a critical dimensional sensitivity of ca. 0.5 nm could be achieved.

## Experimental

Before discussing the electrical contact between the μ4pp electrodes and an individual fin, a general description of a μ4pp measurement on large blanket semiconducting samples is needed. The μ4pp electrodes comprise four Ni-coated Si cantilevers with a spacing of 8 µm and a contact size *d*_contact_ ≈ 300 nm [6,9–10]. In a μ4pp measurement, the electrodes are landed on the sample surface after which a current *I*_in_ is injected into the investigated sample via two of the electrodes while the induced voltage drop *V* is measured between the other two electrodes. Initially, however, the native oxides present both on the semiconducting material and the Ni-coated electrodes act as highly resistive barriers and therefore prevent any electrical contact [11]. To establish the electrical contact, the μ4pp technique uses the so-called punch-through current, i.e., a short current pulse of magnitude *I*_pulse_ applied between two electrodes, which causes the breakdown of the native oxide barrier [12–14] and hence creates the conductive path required to inject *I*_in_ into the investigated material. Empirically, it is observed that the magnitude of *I*_pulse_ must be chosen larger than a certain threshold current (*I*_threshold_, typically >100 µA for blanket materials) in order to reduce the contact resistance *R*_C_ between the electrodes and the sample and hence activate the required electrical contact.

**Figure 1:**
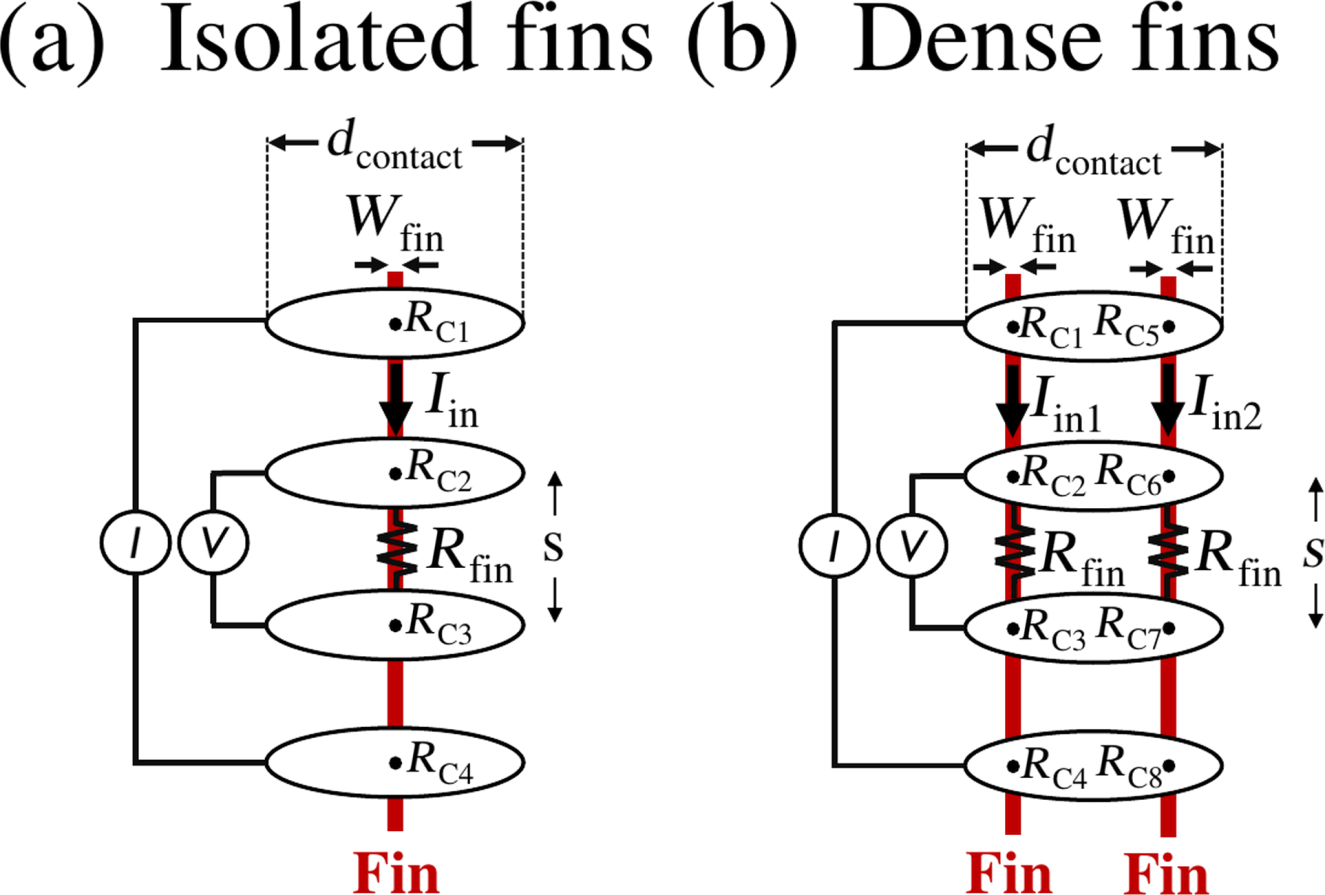
Top-view schematic of the four μ4pp electrodes landed on (a) a single fin and (b) two fins. The electrode contact size and the contact resistance for each electrode–fin contact are, respectively, indicated by *d*_contact_ and *R*_C_*_j_* (*j* = 1, 2,…, 8). Note that all contact resistances are initially considered to be highly resistive because the native oxides present on both the fins and electrodes prevent current flow into the fin. *R*_fin_ is defined as the resistance of the fin between the two inner contacts, i.e., *R*_fin_ = *R_s_* × *s*/*W*_fin_*_,_* where *s* is the distance between the two inner contacts and *W*_fin_ is the fin width.

The given description of the punch-through mechanism is also valid for more confined structures, such as fins. This does, however, require some additional considerations, starting with the distinction between isolated and dense fins. First, for isolated fins (Figure 1a), i.e., fins are separated by a distance (= pitch) larger than *d*_contact_, the procedure is identical to the previously described case of blanket materials. The electrical contact is indeed created, i.e., contacts *j* = 1, 2, 3, 4 are activated, when *I*_pulse_ ≥ *I*_threshold_ and the electrical resistance *R*_fin_ of the region of the fin included between the two inner contacts is readily obtained from the ratio *R*_fin_ *= V*/*I*_in_ [6]. Secondly, in the more complex case of dense fins, i.e., fin pitch < *d*_contact_, the μ4pp electrodes can physically contact multiple fins at the same time. For simplicity, this paper only considers the case of two fins physically contacted by the electrodes (Figure 1b). In this situation, electrical contact is formed on both fins, i.e., contacts *j* = 1, 2, …, 8 are activated, when the magnitude of *I*_pulse_ is similar as used on blanket materials. The measured resistance is then determined by the ratio between the two currents *I*_in1_ and *I*_in2_ injected into the two electrically connected fins. Since this ratio depends on the contact resistances *R*_C_*_j_* (*j* = 1, 4, 5, 8), this leads to a high measurement variability, i.e., a loss in precision [3]. As a consequence, in order to precisely determine *R*_fin_ in a dense fin array, *I*_pulse_ should be carefully controlled (*I*_pulse_ < 2 × *I*_threshold_) to only allow for the formation of electrical contact to one single fin, i.e., only contacts *j* = 1, 2, 3, 4 or *j* = 5, 6, 7, 8 are activated. On top of that, to make sure that all four electrodes indeed form electrical contact with the same fin, the punch-through mechanism between electrode pairs must be sequenced properly. For example, when first applying the punch-through mechanism on the top two electrodes, which then form electrical contact with the left fin in Figure 1b, i.e., contacts *j* = 1 and *j* = 2 are activated, the next electrode pair should use an already activated contact to make sure the contacts on the same fin, i.e., *j* = 3 or *j* = 4, are activated next. Note that, however, the exact behavior of the electrical contact formation on dense fins is still not fully understood and a more thorough description would also include pulse duration, peak voltage, and material properties.

## Results and Discussion

**Figure 2:**
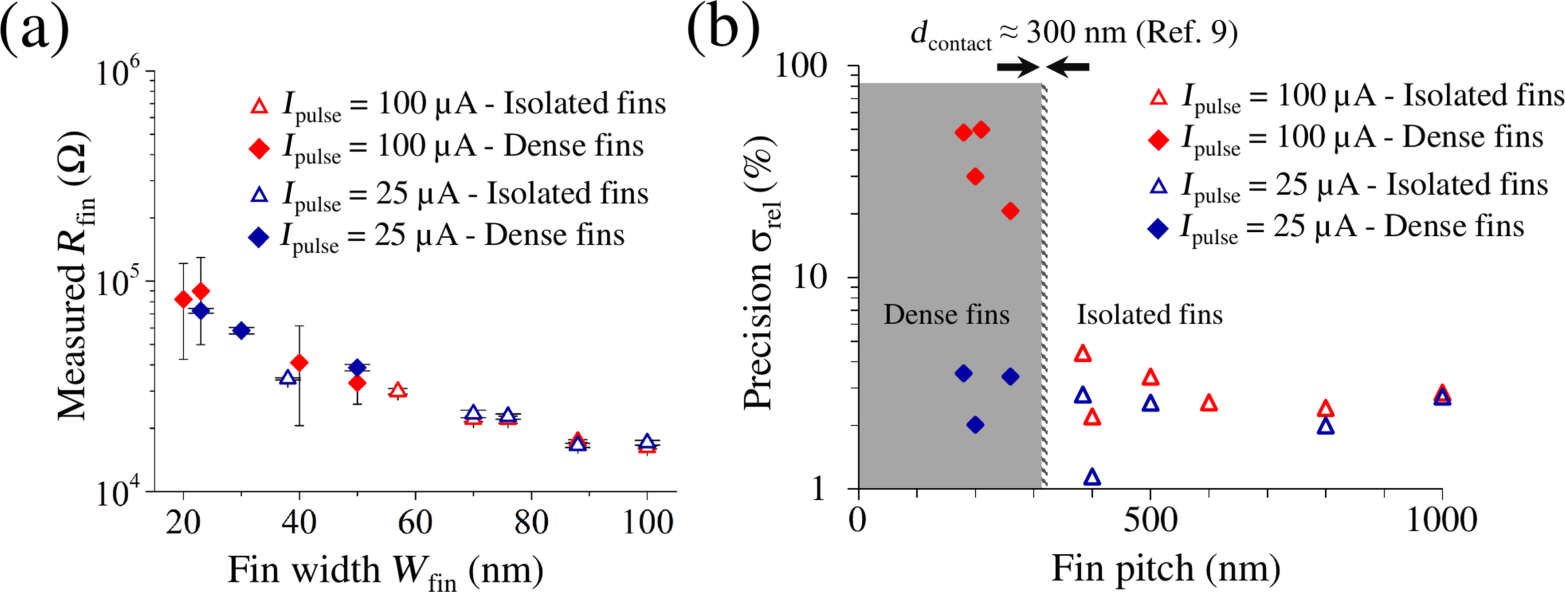
(a) Measured fin resistance *R*_fin_ as a function of fin width *W*_fin_ on isolated (triangle) and dense (diamond) fins using high (red) and low (blue) punch-through currents. (b) Relative standard deviation of the measured values of *R*_fin_ of Figure 2a as a function of the fin pitch. When using a low punch-through current (blue), the relative standard deviation remains stable (≤3%) regardless of fin pitch, indicating that the electrical contact remains restricted to a single fin, even in the grey area where the electrodes are in physical contact with more than one fin.

The experimental demonstration of using the punch-through current *I*_pulse_ to individually contact single Si fins in dense arrays is shown in Figure 2a, where the measured *R*_fin_ is plotted as a function of the fin width *W*_fin_ after using a high (100 µA) or low (25 µA) punch-through current to form the electrical contact. To highlight the impact of the fin pitch, we have additionally separated the isolated and dense fins, assuming the approximately 300 nm physical contact size of the electrodes as measured with scanning electron microscopy (SEM) [9]. It can be observed that, while *I*_pulse_ does not affect the precision on isolated fins (red and blue triangles), for dense fins a major improvement in precision can be achieved by decreasing the punch-through current from 100 μA (red diamonds) to 25 μA (blue diamonds). Based on the previous theoretical considerations, the improvement in precision is achieved by restricting the electrical contact to one single fin despite the electrode being in physical contact with two fins. To show this improvement more clearly, the relative standard deviation of the measured values of *R*_fin_ can be plotted against the fin pitch, as shown in Figure 2b. Excitingly, the precision of the 25µA punch-through current measurement remains stable at around 3%, making the measurement feasible even for fin pitch much smaller than *d*_contact_. Note that Figure 2 also shows that the fin width has no impact on the measurement precision.

**Figure 3:**
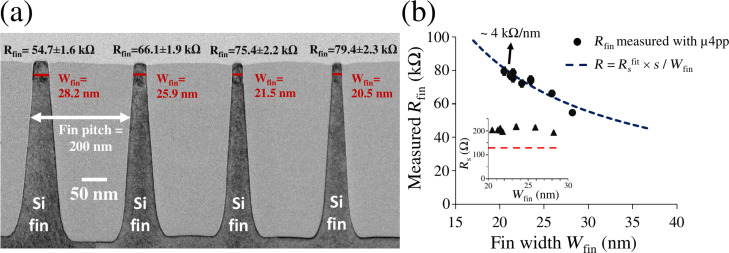
(a) TEM image of four ca. 20 nm wide Si fins where the measured *R*_fin_ is indicated on top of each fin. The measured values of *R*_fin_ correlate to the respective fin width *W*_fin_ according to *R*_fin_ = *R_s_*^fin^ × *s*/*W*_fin_. The error for each fin refers to the lowest precision (3.0%) achieved on all measured fins. (b) Measured fin resistance as a function of *W*_fin_ fitted to a constant sheet resistance *R_s_*^fin^ using the relation *R*_fin_= *R_s_*^fin^ × *s*/*W*_fin_ (using *s* = 8 µm). The slope of the fitted curve at *W*_fin_ = 20 nm is indicated (ca. 4.0 kΩ/nm). (inset) Sheet resistance (*R_s_*^fin^) of the ten Si fins obtained using the inversed relation *R_s_*^fin^ = *R*_fin_ × *W*_fin_/*s*, plotted against fin width *W*_fin_. For comparison, the dashed red line shows the low sheet resistance *R_s_*^pad^ = 135 Ω measured on a large pad of the same material as the fins.

The ability to probe individual fins in dense arrays allows us to exploit the high precision of the μ4pp tool [15] to electrically characterize nanometer-wide fins regardless of the fin pitch. To demonstrate this, Figure 3 shows that we can now measure variations in fin resistance induced by nanometric variations in fin width in a dense array of narrow Si fins. For this, we used an array of ten ca. 20 nm wide Si fins implanted with B (3 × 10^15^ cm^−2^, 5 kV) and laser-annealed three times at 1150 °C. Note that *W*_fin_ is assumed constant, i.e., the very small tapering of the fins along the shallow (ca. 60 nm) implant depth [6] is ignored. These fins, having a pitch of 200 nm, were measured individually by using a punch-through current of 25 µA to restrict the electrical contact to a single fin. Moreover, by running the μ4pp measurement over the fin array with a step size of ca. 25 nm, we could assign the measured values of *R*_fin_ to each specific fin. As can be observed for the four out of ten fins shown in Figure 3a, *R*_fin_ varies in accordance with the fin width measured by TEM. Note that the error in Figure 3a is 3.0% for each fin, which was obtained by taking the lowest precision achieved out of all ten measured fins. Since *R*_fin_ is obtained by taking the average of several subsequent measurements, the precision includes the variation in the exact position of the electrical contact points for each landing of the electrodes, i.e., a variation in contact spacing *s*, which may result both from a variation in the electrode positioning itself and from the exact location of the small electrical contact under the wider electrode. Additionally, using the widths measured with TEM and the relation *R*_fin_ = *R_s_*^fin^ × *s*/*W*_fin_ (using *s* = 8 µm), the inset of Figure 3b shows that all ten fins have the same sheet resistance *R_s_*^fin^ ≈ 200 Ω/sq, indicating that the observed variations in *R*_fin_ are indeed caused by variations in fin width. This allows us to evaluate the sensitivity of the technique by plotting the measured values of *R*_fin_ as a function of the fin width (Figure 3b) and subsequently fitting the data with a constant sheet resistance *R_s_*^fin^ using the relation *R*_fin_ = *R_s_*^fin^ × *s*/*W*_fin_. By comparing the slope of the fitted curve at *W*_fin_ = 20 nm (ca. 4 kΩ/nm) to the achieved precision (ca. 2.3 kΩ, Figure 3a), we can deduce that the technique has a sensitivity to variations in fin width down to about 0.5 nm, opening the prospects for its use in electrical critical dimension metrology. As also interestingly shown in the inset of Figure 3b, the measured *R_s_*^fin^ is higher than the sheet resistance *R_s_*^pad^ = 135 Ω (dashed red line) measured in a large 80 × 80 μm^2^ pad having undergone the same implantation and annealing treatment. This increase in sheet resistance when going to nanoscale elongated geometries was expected and understood to originate from the presence of interface states as well as defects at the fin sidewalls [6].

## Conclusion

This paper demonstrates the capability of μ4pp to electrically characterize individual nanometer-wide Si fins in dense arrays regardless of fin pitch. By carefully controlling the electrical contact, we were able to measure the resistance of individual ca. 20 nm wide fins in dense arrays even though the μ4pp electrodes physically contact more than one fin. Thanks to the high precision of the measurements, the correlation between measured resistance and nanometer-scale variations in fin width could be demonstrated with a sensitivity as small as 0.5 nm.
